# Fatty Acids Composition of Vegetable Oils and Its Contribution to Dietary Energy Intake and Dependence of Cardiovascular Mortality on Dietary Intake of Fatty Acids

**DOI:** 10.3390/ijms160612871

**Published:** 2015-06-05

**Authors:** Jana Orsavova, Ladislava Misurcova, Jarmila Vavra Ambrozova, Robert Vicha, Jiri Mlcek

**Affiliations:** 1 Language Centre, Faculty of Humanities, Tomas Bata University in Zlín, nám. T. G. Masaryka 5555, 760 01 Zlín, Czech Republic; E-Mail: orsavova@fhs.utb.cz; 2 Department of Food Analysis and Chemistry, Faculty of Technology, Tomas Bata University in Zlín, nám. T. G. Masaryka 5555, 760 01 Zlín, Czech Republic; E-Mails: ambrozova@ft.utb.cz (J.V.A.); mlcek@ft.utb.cz (J.M.); 3 Department of Chemistry, Faculty of Technology, Tomas Bata University in Zlín, nám. T. G. Masaryka 5555, 760 01 Zlín, Czech Republic; E-Mail: rvicha@ft.utb.cz

**Keywords:** vegetable oils, fatty acids, cardiovascular diseases, coronary heart diseases, Spearman’s correlation

## Abstract

Characterizations of fatty acids composition in % of total methylester of fatty acids (FAMEs) of fourteen vegetable oils—safflower, grape, *silybum marianum*, hemp, sunflower, wheat germ, pumpkin seed, sesame, rice bran, almond, rapeseed, peanut, olive, and coconut oil—were obtained by using gas chromatography (GC). Saturated (SFA), monounsaturated (MUFA) and polyunsaturated fatty acids (PUFA), palmitic acid (C16:0; 4.6%–20.0%), oleic acid (C18:1; 6.2%–71.1%) and linoleic acid (C18:2; 1.6%–79%), respectively, were found predominant. The nutritional aspect of analyzed oils was evaluated by determination of the energy contribution of SFAs (19.4%–695.7% E_RDI_), PUFAs (10.6%–786.8% E_RDI_), n-3 FAs (4.4%–117.1% E_RDI_) and n-6 FAs (1.8%–959.2% E_RDI_), expressed in % E_RDI_ of 1 g oil to energy recommended dietary intakes (E_RDI_) for total fat (E_RDI_—37.7 kJ/g). The significant relationship between the reported data of total fat, SFAs, MUFAs and PUFAs intakes (% E_RDI_) for adults and mortality caused by coronary heart diseases (CHD) and cardiovascular diseases (CVD) in twelve countries has not been confirmed by Spearman’s correlations.

## 1. Introduction

Lipids are considered one of the most elemental nutrients for humans. Lipid metabolism generates many bioactive lipid molecules, which are fundamental mediators of multiple signaling pathways and they are also indispensable compounds of cell membranes. Any kind of changes in lipid metabolism can result in modification of membrane composition and subsequently in changes in its permeability. It may also lead to disruption of signaling networks and could be associated with some pathological states, such as cancer, cardiovascular, neurodegenerative, and metabolic diseases, and similarly with inflammatory complications [1,2,3,4,5,6,7,8,9]. Lipids consist of fatty acids (FAs) classified mostly according to the presence or absence of double bonds as saturated (SFAs—without double bonds), monounsaturated (MUFAs—with one double bond) and polyunsaturated fatty acids (PUFAs—with two or up to six double bonds); further, as *cis* or *trans* based on the configuration of the double bonds and as n-3 or n-6 PUFAs depending on the position of the first double bond from the fatty acid methyl-end. The human body cannot synthesize PUFAs with the first double bond on C3 and C6 from the methyl-end because of the absence of appropriate enzymes. Thus, these fatty acids are essential (EFAs) and they have to be obtained from a diet, particularly by the consumption of fish and fish oils [1,2,3,4,5,10].

Unsaturated fatty acids can exist in a *cis-* or *trans-*configuration. The former configuration is found in most naturally occurring unsaturated fatty acids, the latter configuration is the result of technology processing, such as hydrogenation. *Cis*-unsaturated fatty acids are potent inducers of adiposomes known as lipid droplets, which have important roles in cell signaling, regulation of lipid metabolism and control of the synthesis and secretion of inflammatory mediators. Lipid droplets are sites for eicosanoid generation in cells during a process of inflammation and cancer [11].

Fundamental PUFAs are α-linolenic (ALA, 18:3, n-3) and linoleic acid (LA, 18:2, n-6) from which other important PUFAs are derived [1]. Satisfactory transformation of ALA to docosahexaenoic acid (DHA, 22:6, n-3) depends on the activity of responsible ∆^5^ and ∆^6^ desaturases that could be influenced by several factors, such as dietary cholesterol and high-fat diet and also appears to be low in diabetics [12,13,14]. Further, decreasing effects on the ∆^6^ desaturase activity and therefore the conversions of LA and ALA to long chained polyunsaturated fatty acids (LCPUFAs) caused by low insulin levels, deficiency of protein and minerals, such as iron, zinc, copper, and magnesium have also been published [15]. Furthermore, conversion of dietary ALA into eicosapentaenoic acid (EPA, 20:5, n-3) is limited because of the competition for common desaturation and elongation enzymes of ALA and LA. Moreover, it has been proved that the affinity of ∆^6^ desaturase for ALA is greater than for LA [3].

Recently, essential fatty acids (EFAs) have been considered as functional food and nutraceuticals. A lot of research studies have documented their significant roles in many biochemical pathways resulting in cardioprotective effect because of their considerable antiatherogenic, antithrombotic, anti-inflammatory, antiarrhytmic, hypolipidemic effect, because of the potential of reducing the risk of serious diseases, especially cardiovascular diseases, cancer, osteoporosis, diabetes and other health promotion activities following from their complex influence on concentrations of lipoproteins, fluidity of biological membranes, function of membraned enzymes and receptors, modulation of eicosanoids production, blood pressure regulation, and finally, on the metabolism of minerals [1,16,17,18,19,20,21]. EPA and DHA have also been associated with the protection against mental disorders like Alzheimer’s disease, aging and dementia, chronic daily headache and with attention-deficit hyperactivity disorder in children [22,23,24]. Biological activities of individual EFAs might be derived from the character and three-dimensional configuration of molecules and their subsequent enzymatic transformation in a wide scale of compounds named eicosanoids. Eicosanoids derived from n-6 and n-3 fatty acids have antagonistic effects. Eicosanoids from the first group promote an inflammation; the latter are much less inflammatory or even anti-inflammatory. Their concentration depends on the fatty acids amounts in diet and is also influenced by the competition between AA and EPA as substrates for specific enzymes—cyclooxygenases and 5-lipoxygenases [1,18,25].

Seriously, cardiovascular diseases have been documented to be the main cause of death in most Western countries. Coronary heart disease is closely connected with a progress of atherosclerosis evoked by the interactions between plasma lipids, lipoproteins, monocytes, platelets, endothelium, and smooth muscle of arterial walls resulting in narrowed coronary arteries [1]. Thus, dietary modulation with emphasis on the composition of dietary lipids could be a therapeutic option in the prevention of thrombosis and coronary infarctions and in the treatment of various diseases including hearth diseases to improve the quality of arterial walls and vascular patency. The important role of dietary pattern and lifestyle on human health has been often documented. 1.5 times higher blood level of trans fatty acids in younger Inuit compared to elder Inuit was observed in connection with a decrease in traditional food consumption accompanied with an increased consumption of supermarket food, *i.e.*, Western diet [10]. Recently, nutritionists have recommended vegetable oils as an important part of a healthy diet due to their high contents of fatty acids (FAs) besides their traditional sources, such as fish oil and algae [1,26,27,28]. However, distribution and content of fatty acids differ in dependence on various plant sources of oils and technology process used for their production.

This paper evaluated FAs composition of some vegetable oils, energy contribution E (% E_RDI_) of saturated (SFAs), polyunsaturated (PUFAs) fatty acids, n-3 PUFAs and n-6 PUFAs of analyzed oils to recommended dietary intakes for total fat (E_RDI_—37.7 kJ/g). The amounts (g) of SFAs, PUFAs, n-3 PUFAs and n-6 PUFAs requisite to cover their recommended daily intakes were calculated using maximal values of recommended daily intakes for fatty acids (E_RDI_). Statistic evaluation of the relationship between reported data of total fat, SFAs, MUFAs and PUFAs intakes among adults in different countries and coronary heart diseases (CHD) and cardiovascular diseases (CVD) mortality by Spearman’s correlations were computed.

## 2. Results and Discussion

### 2.1. Fatty Acids Composition of Vegetable Oils

Fourteen samples of analyzed vegetable oils were mostly produced as virgin oils with a sustentative content of monounsaturated (MUFAs) and polyunsaturated (PUFAs) fatty acids. They have been considered as functional food and used as an important part of a healthy diet. Their FAs compositions are presented in Table 1.

**Table 1:** Fatty acids composition of vegetable oils ^1^.

FAs [%]	SAF	GRP	SIL	HMP	SFL	WHG	PMS	SES	RB	ALM	RPS	PNT	OL	COC
C6:0	nd	nd	nd	nd	nd	nd	nd	nd	nd	nd	nd	nd	nd	0.52
C8:0	nd	0.01	nd	nd	nd	nd	nd	nd	nd	nd	nd	nd	nd	7.6
C10:0	nd	nd	nd	nd	nd	nd	nd	nd	nd	nd	0.01	nd	nd	5.5
C12:0	nd	0.01	0.01	nd	0.02	0.07	nd	nd	nd	0.09	nd	nd	nd	47.7
C14:0	0.10	0.05	0.09	0.07	0.09	nd	0.17	nd	0.39	0.07	nd	0.04	nd	19.9
C15:0	nd	0.01	0.02	nd	nd	0.04	nd	nd	nd	nd	0.02	nd	nd	nd
C16:0	6.7	6.6	7.9	6.4	6.2	17.4	13.1	9.7	20.0	6.8	4.6	7.5	16.5	nd
C17:0	0.04	0.06	0.06	0.05	0.02	0.03	0.13	nd	nd	0.05	0.04	0.07	nd	nd
C18:0	2.4	3.5	4.5	2.6	2.8	0.7	5.7	6.5	2.1	2.3	1.7	2.1	2.3	2.7
C20:0	nd	0.16	2.6	nd	0.21	nd	0.47	0.63	nd	0.09	nd	1.01	0.43	nd
C22:0	nd	nd	nd	nd	nd	nd	nd	0.14	nd	nd	nd	nd	0.15	nd
C16:1 (n-7)	0.08	0.08	0.05	0.11	0.12	0.21	0.12	0.11	0.19	0.53	0.21	0.07	1.8	nd
C17:1 (n-7)	nd	nd	0.03	nd	nd	nd	nd	nd	nd	nd	nd	nd	nd	nd
C18:1cis (n-9)	11.5	14.3	20.4	11.5	28.0	12.7	24.9	41.5	42.7	67.2	63.3	71.1	66.4	6.2
C18:1trans (n-9)	nd	nd	nd	nd	nd	nd	nd	nd	nd	nd	0.14	nd	nd	nd
C20:1(n-9)	nd	0.40	0.15	16.5	0.18	7.91	1.08	0.32	1.11	0.16	9.1	nd	0.30	nd
C18:2cis (n-6)	79.0	74.7	63.3	59.4	62.2	59.7	54.2	40.9	33.1	22.8	19.6	18.2	16.4	1.6
C18:3 (n-3)	0.15	0.15	0.88	0.36	0.16	1.2	0.12	0.21	0.45	nd	1.2	nd	1.6	nd
C18:3 (n-6)	nd	nd	nd	3.0	nd	nd	nd	nd	nd	nd	nd	nd	nd	nd
SFAs	9.3	10.4	15.1	9.2	9.4	18.2	19.6	16.9	22.5	9.3	6.3	10.7	19.4	92.1
MUFAs	11.6	14.8	20.7	28.1	28.3	20.9	26.1	42.0	44.0	67.9	72.8	71.1	68.2	6.2
PUFAs	79.1	74.9	64.2	62.8	62.4	61.0	54.3	41.2	33.6	22.8	20.9	18.2	18.0	1.6
n-3 PUFAs	0.2	0.2	0.9	0.4	0.2	1.2	0.1	0.2	0.5	0.0	1.2	0.0	1.6	0.0
n-6 PUFAs	79.0	74.7	63.3	62.4	62.2	59.7	54.2	40.9	33.1	22.8	19.6	18.2	16.4	1.6

^1^ Data are expressed as percentages of total fatty acid methyl esters (FAMEs); nd means that FAs was not determined. Abbreviations of the samples mean: SAF—safflower, GRP—grape, SIL—*silybum marianum*, HMP—hemp, SFL—sunflower, WHG—wheat germ, PMS—pumpkin seed, SES—sesame, RB—rice bran, ALM—almond, RPS—rapeseed, PNT—peanut, OL—olive, and COC—coconut oils.

Fatty acids composition of vegetable oils is formed by a mixture of saturated (SFAs) and unsaturated (UNFAs) fatty acids classified according to the number of unsaturated bonds as monounsaturated (MUFAs) or polyunsaturated fatty acids (PUFAs). Nevertheless, each of analyzed vegetable oils has specific fatty acid distribution depending on their plant sources. So, their impact on human health could be assessed according to individual fatty acids because of their different influences on human health and risks of serious diseases.

#### 2.1.1. Saturated Fatty Acids (SFAs)

Saturated fatty acids with fewer than 12 carbon atoms being called short and medium chain saturated fatty acids (MCFAs) have been found only in coconut oil (COC) in the amount not exceeding 7.6% of total methylester of fatty acids (FAMEs) as can be seen in Table 1. Expectedly, SFAs were established as extraordinarily predominant FAs in the highest amount of 92.1% of total FAMEs in coconut oil (COC), and they were presented especially by lauric (C12:0) and myristic (C14:0) acids in the amounts of 47.7% and 19.9%, respectively; in agreement with reported data [18,25,29,30,31,32]. In the rest of analyzed oils SFAs were determined in the range from 6.3% (rapeseed oil (RPS)) to 22.5% (rice brain oil (RB)) of total FAMEs. Palmitic acid (C16:0) was found to be a predominant SFA in the majority of samples in the range from 4.6% (RPS) to 20.0% (RB). In short, observed contents of palmitic acid (C16:0) were also in agreement with reported data by [30,31,32,33,34,35,36,37].

In fact, some studies have reported various impacts of SFAs on the human health. It has been concluded that lauric acid (C12:0) as well as myristic acid (C14:0) raise plasma total cholesterol concentrations, the first due to an increase in LDL cholesterol, the latter due to a rise of both LDL and HDL cholesterol concentrations [38,39]. However, according to Mensink [40] and Lawrence [41], the ratio of total cholesterol to HDL cholesterol is a more specific marker of coronary artery diseases than the value of LDL cholesterol. Oils rich in lauric acid (C12:0) decreased the ratio of total to HDL cholesterol. On the other hand, myristic (C14:0) and palmitic acids (C16:0) affected this ratio only little and stearic acid (C18:0) slightly reduced this ratio.

#### 2.1.2. Monounsaturated Fatty Acids (MUFAs)

The Mediterranean diet is well-known as a diet with high consumption of olive oil and minimal amount of saturated fatty acids. Red meat, whole fat milk products, nuts and high fat fruits, such as olives and avocados are among the natural sources of MUFAs. The majority of all investigated samples, except for coconut oil, showed the highest proportion of MUFAs or PUFAs in their FAMEs composition. In general, MUFAs were distributed mostly in higher amounts than SFAs in the range from 6.2% to 72.8% in coconut (COC) and rapeseed (RPS) oils, respectively. Interestingly, MUFAs formed a main part of fatty acid compositions in six analyzed oils, such as 72.8% in rapeseed oil (RPS), 71.1% in peanut oil (PNT), 68.2% in olive oil (OL), 67.9% in almond oil (ALM), 44.0% in rice brain oil (RB), and 42.0% in sesame oil (SES). Oleic acid (C18:1, n-9) was found as the most abundant MUFA in almost all samples in the range from 6.2% (COC) to 71.1% (PNT), except for hemp oil (HMP), where eicosenoic acid (C20:1, n-9) was established as the predominant MUFA in the amount of 16.5% conversely to reported data by [30], where eicosenoic acid in hemp oil was not determined. Observed contents of oleic acid in the selected samples of vegetable oils were significantly in accordance with reported values [30,31,32,33,34,35,36,37] with the exception of oleic acid content in peanut oil (PNT) that was higher in comparison to reported data [30,32]. It has been documented that MUFAs may reduce LDL cholesterol, while it might possibly increase high-density lipoprotein (HDL) cholesterol [15]. Oleic acid (C18:1, n-9) may promote insulin resistance contrary to PUFAs with the protection against insulin resistance [15]. Further, the saturation index (SI) in red blood cell membranes is formed by the ratio of stearic (C18:0, SFA) to oleic acid (C18:1, n-9, MUFA) and it is found as an appropriate biomarker for investigating the relation between the pattern of metabolism and breast cancer risk [42]. Oleic acid has also been reported as anti-apoptotic and anti-inflammatory agent via down regulation of cyclooxygenase-2 (COX-2) and inducible nitric oxide synthase (iNOS) through the activation of nuclear factor-kappa B (NF-κB) resulting in the activation of downstream inflammatory mediators [43].

#### 2.1.3. Polyunsaturated Fatty Acids (PUFAs)

Primary source of PUFAs, especially docosahexaenoic acid (DHA, C22:6, n-3) and linoleic acid (LA, C18:2, n-6) is algae and marine phytoplankton forming the main part of fish feed as was previously described by [1,44,45,46]. Terrestrial sources are found mostly in nuts, seeds, and leafy vegetables [47]. In seven analyzed oils—safflower oil (SAF), grape oil (GRP), *silybum marianum* oil (SIL), hemp oil (HMP), sunflower oil (SFL), wheat germ (WGM), and pumpkin seed oil (PMS), PUFAs presented as the predominant part of fatty acid compositions and their contents ranged from 54.3% in pumpkin seed oil (PMS) to 79.1% in safflower oil (SAF). The most abundant PUFA was linoleic acid (LA, C18:2, n-6) in all analyzed samples, in the range from 1.6% in coconut oil (COC) to 79.0% in safflower oil (SAF). Similar results of LA (C18:2, n-6) have been reported for grape, almond, wheat germ, sesame, pumpkin seed and safflower oil [33]; for peanut, rapeseed and coconut oil [30,32]; and finally for hemp oil [30], rice brain oil [31], sunflower oil [31,37] and olive oil [37,48]. However, the obtained content of 63.3% of LA (C18:2, n-6) in SIL was higher than the published amount [35].

Recent studies have clearly shown the important impact of polyunsaturated fatty acids (PUFAs) on human health in the prevention of, particularly, cardiovascular disease (DVD), coronary heart disease and cancer; further, inflammatory, thrombotic and autoimmune disease; hypertension; diabetes type two, renal diseases; and rheumatoid arthritis, ulcerative colitis, and Crohn’s disease. Their non-substitutable roles in many biological pathways are crucial [49,50].

The difference between the locations of the first double bond in the fatty acid carbon chain (n-3 and n-6 PUFAs) is the reason of significant differences in their biological functions that might be derived from the course of their interactions [1]. The n-6/n-3 ratio is considered to be the key factor for balanced synthesis of eicosanoids and its nutritional importance has been frequently discussed as well as dependence of n-6/n-3 ratio value on a dietary pattern. High consumption of plant oils rich in n-6 PUFAs and relatively low consumption of marine fish products could cause this ratio to be too high, mainly seen in Western countries. Though, industrially produced food with high content of n-6 PUFAs also acquired popularity in the regions with traditional dietary patterns characterized by low blood value of n-6/n-3 ratio [1,10,49]. In the analyzed oils, n-3 PUFAs represented by α-linolenic acid (ALA, C18:3, n-3) were found in the range of 0.1%–1.6%, except for PNT, ALM and COC oils, where n-3 PUFAs were not determined. The group of n-6 PUFAs was represented by linoleic (LA, C18:2, n-6) and γ-linolenic (GLA, C18:3, n-6) acids. Whereas LA was found to be the predominant PUFAs in the analyzed samples in the range of 1.6%–79.0%, GLA was found only in sHMP in the amount of 3.0%.

### 2.2. Contribution of Fatty Acid Groups of Vegetable Oils to Energy Daily Intake

Lipids belong to main sources of energy for human metabolic processes. Lipid consumption in most Western countries is relatively high with the contribution of approximately 40% of total calories [51]. This is despite the nutritional recommendation that 25% of energy should be covered by lipids [52] and total fat daily intake of 20%–35% of energy is recommended by Food and Agriculture Organization (FAO) of the United Nations and the World Health Organization (WHO) [15]. Nevertheless, variations in the structures of individual fatty acids appear to elicit their different physiological functions with diverse impacts on human health. However, the absolute dietary intake of lipids is not considered as the main promoter of cardiovascular diseases. Relative concentration and distribution of fatty acids in dietary fats have been reported to be an important factor in considering nutritional values of lipids as well as the key factor, with proved effects, of lowering the risk of cardiovascular diseases [1,53]. In Table 2 the energy values (kJ/g) calculated from SFAs, MUFAs, PUFAs, n-3 PUFAs and n-6 PUFAs contents of fourteen vegetable oils using the conversion factor of 37.7 kJ/g for fat and fatty acids are shown.

**Table 2:** Energy values (E, kJ/g) of different fatty acid groups derived from 1 g of vegetable oils ^1^.

E (kJ/g)	SAF	GRP	SIL	HMP	SFL	WHG	PMS	SES	RB	ALM	RPS	PNT	OL	COC
SFAs	2.9	2.6	5.0	3.0	4.7	4.1	4.6	3.5	6.2	2.1	4.4	3.2	0.7	26.2
MUFAs	3.7	3.8	6.0	9.5	14.6	4.8	8.9	8.1	12.3	16.4	47.4	22.0	2.6	1.7
PUFAs	26.0	19.7	19.0	21.1	32.6	14.2	12.5	9.4	9.4	5.5	13.6	5.7	0.7	0.4
n-3 PUFAs	0.1	0.0	0.4	0.1	0.1	0.3	0.0	0.0	0.1	0.0	0.9	0.0	0.0	0.0
n-6 PUFAs	25.9	19.6	18.6	20.9	32.5	13.9	12.4	9.4	9.3	5.5	12.8	5.7	0.1	0.4

^1^ Data are expressed as energy values (E) in kJ/g of different fatty acid groups of SFAs, MUFAs, PUFAs, n-3 and n-6 PUFAs derived from 1g of analyzed oils.

Moreover, for an easy evaluation of nutritional aspect of analyzed vegetable oils from the energy contribution E (% E_RDI_) of their SFAs, PUFAs, n-3 PUFAs and n-6 PUFAs point of view, the energy values (kJ/g oil) from Table 2 were converted into the percentages of recommended daily intakes for total fat (E_RDI_—37.7 kJ/g) for different fatty acid groups by using the maximal values of recommended daily intakes of E_RDI–SFAs_—10% E_RDI_; E_RDI–PUFAs_—11% E_RDI_; E_RDI–n-3_—2% E_RDI_ and E_RDI–n-6_—9% E_RDI_ according to FAO/WHO [15]. Data are shown in Table 3.

**Table 3:** Percentage contribution of energy (E) of SFAs, PUFAs, n-3 PUFAs and n-6 PUFAs of vegetable oils to recommended daily intakes for total fat (E_RDI_—37.7 kJ/g) ^1^.

E [% E_RDI_]	SAF	GRP	SIL	HMP	SFL	WHG	PMS	SES	RB	ALM	RPS	PNT	OL	COC
SFAs	77.2	69.9	132.2	78.5	124.9	107.5	121.3	93.9	163.4	56.7	116.2	84.2	19.4	695.7
PUFAs	626.3	474.5	457.1	507.9	786.8	342.3	300.9	226.9	227.2	132.7	329.1	137.4	16.4	10.6
n-3 PUFAs	6.7	5.5	47.8	17.0	11.2	41.1	5.0	4.4	17.6	0.0	117.1	0.0	0.0	0.0
n-6 PUFAs	764.0	578.7	547.6	617.0	959.2	409.3	366.6	276.4	273.8	162.2	376.2	167.9	1.8	13.0

^1^ Data are expressed as percentages of recommended daily intakes for total fat (E_RDI_—37.7 kJ/g) calculated by using maximal values of recommended daily intakes of E_RDI–SFAs_—10% E_RDI_; E_RDI–PUFAs_—11% E_RDI_; E_RDI–n-3 PUFAs_—2% E_RDI_ and E_RDI–n-6 PUFAs_—9% E_RDI_.

According to the recommendations of the Report of an expert consultation of FAO/WHO [15] for adults, total intake of SFAs should not exceed 10% E_RDI_. Recommended MUFAs daily intake has not been given by definite number, but it is calculated from the difference between total fat intake (% E_RDI_) and the amounts of SFAs, PUFAs, and *trans*-fatty acids (TFAs) in % E_RDI_. Further, recommended daily energy intake of total PUFAs is in the range of 6%–11% E_RDI_. Total n-3 PUFAs energy intake should be in the range of 0.5%–2% E_RDI_. Total n-6 PUFAs energy intake is 2.5%–9% E_RDI_, and finally for TFAs, daily energy intake is lesser than 1% E_RDI_ [15].

From results given in Table 3 could be concluded that energy equivalents corresponding SFAs, PUFAs, n-3 PUFAs and n-6 PUFAs energy intakes derived from 1 g of almost all analyzed oils exceeded general recommendations of the Report of an expert consultation of FAO/WHO for adults [15]. Energy values of SFAs ranged from 19.4% E_RDI_ (OL) to 695.7% E_RDI_ (COC). Warningly, the highest value of energy derived from SAFs in 1 g of COC exceeded the recommended maximum of 10% E_RDI_ by almost seventy times. Thus, despite the production of new food products containing coconut oil, its extensive consumption has not been recommended from the view of the hypercholesterolemic effect.

Due to the fact that recommended MUFAs intake has not been given by definite number, energy originating from MUFAs is not included in Table 3.

Similar results of energy values of PUFAs were obtained; they varied between 10.6% E_RDI_ (COC) and 786.8% E_RDI_ (SFL). Importantly, analyzed oil samples represent an abundant source of PUFAs as it is evident from obtained results. Energy contribution of PUFAs derived from 1 g oils in the majority of samples multiply exceeded recommended value of 11% E_RDI_ except for OL with a slightly higher amount of 16.4% E_RDI_ and also COC with a value of 10.6% E_RDI_. Enormous providers of energy derived from PUFAs, besides SFL mentioned above, are also these oils: SAF (626.3% E_RDI_), HMP (507.9% E_RDI_), GRP (474.5% E_RDI_), SIL (457.1% E_RDI_), WHG (342.3% E_RDI_), RPS (329.1% E_RDI_), PMS (300.9% E_RDI_), RB (227.2% E_RDI_) and SES (226.9% E_RDI_). Energy values of PNT and ALM performed 137.4% E_RDI_ and 132.7% E_RDI_, respectively. Since the energy intake from PUFAs higher than 11% E_RDI_ could be a potential risk of lipid peroxidation, the consumption of these oils should be rather in minor amounts and less frequent.

Energy values derived from the last two groups of n-3 PUFAs and n-6 PUFAs also exceeded general recommendations of their energy intakes derived from 1 g oils except for ALM, PNT, OL and COC, where n-3 PUFAs were not determined. Further, if compared to FAO/WHO recommendation for ALA intake of 0.5%–2% E_RDI_ for adults, established amounts of α-linolenic acid (ALA, C18:3, n-3) as the main representative of n-3 PUFAs in the rest of oils showed higher energy contribution in the range from 4.4% E_RDI_ in SES to 117.1% E_RDI_ in RPS. However, energy value of other oils did not exceed 50% E_RDI_—47.8% E_RDI_ in SIL and 41.1% E_RDI_ in WHG.

Contrary to n-3 PUFAs, large contribution of n-6 PUFAs to energy intake has been revealed in the range from 1.8% E_RDI_ (OL) to 959.2% E_RDI_ (SFL). According to FAO/WHO, recommended intake of significant n-6 PUFA—LA for adults is 2.5%–9% E_RDI_. With the only exception of OL, in all other analyzed oils LA multiple exceeded this recommended amount, e.g., 764.0% E_RDI_ (SAF), 617.0% E_RDI_ (HMP), 578.7% E_RDI_ (GRP), 547.6% E_RDI_ (SIL), and 409.3% E_RDI_ (WHG), which is in contrast with insignificant contribution to energy intake of n-6 PUFAs of OL and 13% E_RDI_ (COC) mentioned above.

*Trans* elaidic acid (C18:1, n-9) is the principal *trans*-unsaturated fatty acid often found in partially hydrogenated vegetable oils [54]. It was determined only in RPS and its energy contribution represented a higher amount of 2.7% E_RDI_ in relation with recommended daily intake of less than 1% E_RDI_ for TFAs (result was not shown in Table 3).

### 2.3. Oil Amounts Covering Recommended Daily Intakes of SFAs, PUFAs, n-3 and n-6 PUFAs

**Figure 1:**
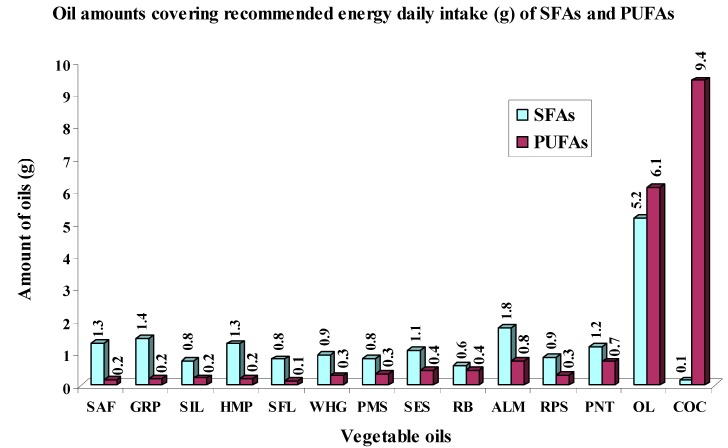
Oil amounts (g) needed to cover recommended energy daily intakes of SFAs and PUFAs calculated from their maximal recommended daily values (E_RDI_).

**Figure 2:**
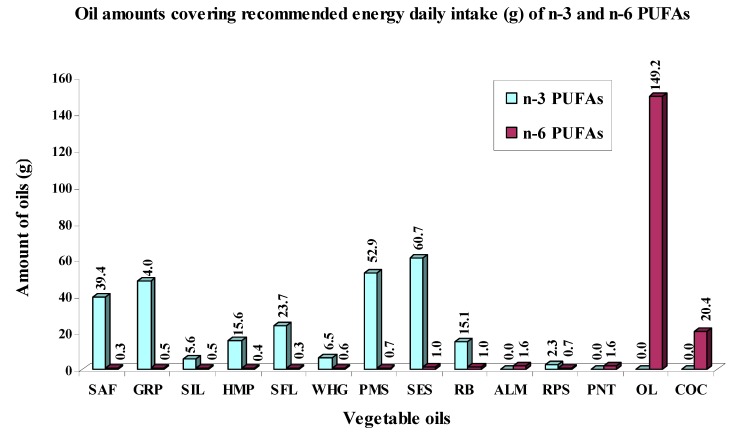
Oil amounts (g) needed to cover recommended energy daily intakes of n-3 PUFAs and n-6 PUFAs calculated from their maximal recommended daily values (E_RDI_).

It is evident that the amounts of analyzed oils for fulfillment of recommended energy daily intakes of individual FAs groups vary according to their specific FAs composition depending on specific plant source. In Figure 1 and Figure 2 are shown the amounts (g) of analyzed vegetable oils that is needed to cover recommended energy daily intakes of SFAs and PUFAs (Figure 1) and further recommended energy daily intakes of n-3 PUFAs and n-6 PUFAs (Figure 2).

For coverage of SFAs energy recommended daily intake are needed oil amounts in the range from 0.1 g (COC) to 5.2 g (OL). Since the analyzed oils were mostly established as important sources of PUFAs, the amounts needed to cover their recommended energy daily intake are relatively low and in the majority of oils it ranged between 0.1 g (SFL) and 0.8 g (ALM) with the exception of the amount of 9.4 g and 6.1 g in COC and OL that are both an abundant source of SFAs and MUFAs.

Importantly, analyzed oils were abundant in contents of n-6 PUFAs, so the amounts needed to cover their recommended daily intakes would have to be small and ranged from 0.3 g (SFL and SAF) to 1.6 g (PNT and ALM). With the exception of OL rich in oleic acid (MUFAs) whose amount of 20.4 g is needed to cover recommended energy daily intake of n-6 PUFAs and COC, which is an abundant source of SFAs, but deficient in n-6 PUFAs, so the amount to cover recommended energy daily intake for n-6 PUFAs is 149.2 g.

Finally, analyzed oils were characterized by mostly a small content of n-3 PUFAs, thus the amounts needed to cover the recommended daily intakes were relatively large from 2.3 g (RPS) to 60.7 g (SES).

### 2.4. Statistical Evaluation of Impact of Fat Dietary Intake on the Number of CHD and CVD Mortality in Various Countries

The role of dietary fats in cardiovascular disease (CVD) and many other disorders has been often discussed with general conclusions that this process is not simple, but rather a consequence of complex factors. Nevertheless, each group of fatty acids—SFAs, MUFAs, PUFAs and individual FAs—has a specific role in many biopathways and imbalance in their dietary intake could be result in many serious diseases. Coronary heart diseases (CHD) induce the biggest mortality worldwide. The major risk factors are contemporary lifestyle characteristic in physical training deficit, smoking, excessive food intake and wrong dietary pattern leading to overweight or obesity, high blood pressure, high level of total cholesterol, LDL cholesterol, and triglycerides [55]. In this section, statistical evaluation of impact of fat dietary intakes on CHD and CVD mortality in various countries is provided by using coronary heart disease (CHD) number and cardiovascular disease (CVD) mortality per 100,000 inhabitants and intakes of total fats, SFAs, MUFAs, and PUFAs of an adult in different countries. Data about CHD and CVD mortality were collected from the sources [56,57,58,59,60] and intakes of total fats, SFAs, MUFAs and PUFAs in different countries were used according to [61], all shown in Table 4.

As seen in Table 4, it could be concluded different intakes (% E_RDI_) of total fats, SFAs, MUFAs and PUFAs in some European countries (the Czech Republic, Germany, Austria, Finland, United Kingdom, Norway, France and Greece), the USA, the Republic of South Africa, Australia with New Zealand, and Japan. Generally, a lower intake of SFAs was mostly in relation with a lower intake of total fat. The highest intakes of SFAs in some European countries and the USA could give evidence to popularity of hydrogenated fats intakes; similarly, the highest MUFAs intake in Greece is probably in connection with the Mediterranean dietary pattern characterized by high consumption of olive oil. Documented intakes of PUFAs are very heterogeneous in the range of 3.9%–8.0% E_RDI_ and are not in relation with number of CHD and CVD mortality in monitored countries. In accordance with data from Table 4, relatively high intakes of total fats were documented in global perspective study by Elmadfa and Kornsteiner [55] in Europe in the range of 28.5%–46.2% E_RDI_, but also in Africa 13.1%–50.7% E_RDI_, and America 25.7%–37.2% E_RDI_. In the other continents—Asia and Australia—they were in the ranges of 11.1%–35.6% E_RDI_ and 32.5%–35.0% E_RDI_, respectively. According to [55], the highest intakes of MUFAs 10.9%–22.3% E_RDI_ were also reported in Europe, further in Australia in the range of 11.8%–12.0% E_RDI_, while the highest PUFAs intakes were 3.3%–11.3% E_RDI_ in Asia, which could be a consequence of typical cuisine patterns with high consumption of fish oil with the high content of PUFAs.

**Table 4:** CHD and CVD mortality and intakes of total fats, SFAs, MUFAs and PUFAs of an adult in various countries.

Country	CHD + Other CVD Mortality Per 100,000 ^1^	Total Fats	SFAs	MUFAs	PUFAs
		(% E_RDI_) ^2^			
Czech Republic	388.27	36.0	13.0	13.0	7.0
Germany	364.75	35.9	14.4	12.8	6.5
Austria	320.92	37.0	14.5	12.5	8.0
Finland	284.69	32.1	13.5	12.4	6.2
USA	260.58	34.0	11.0	12.5	7.0
United Kingdom	243.45	32.9	12.0	11.7	5.9
Norway	242.72	30.6	12.1	10.8	5.4
Republic of South Africa	237.45	27.7	8.6	9.5	6.9
France	181.40	37.2	14.1	11.8	3.9
Australia, New Zealand	162.44	33.1	12.9	12.1	5.0
Greece	141.04	46.2	13.1	22.3	6.6
Japan	28.00	25.3	8.4	9.4	7.5

^1^ Data were collected from the sources [56,57,58,59,60]; ^2^ Data of total fats, SFAs, MUFAs and PUFAs intakes in different countries were used according to [61].

Relatively high number of CHD and CVD mortality has been found in some European countries, especially in the Czech Republic, Germany and Austria, with values exceeding three hundred events per 100,000 habitants. However, not only total fat intakes, but other factors, such as dietary pattern and stressful life style, and amounts and distribution of fatty acids could contribute to CHD and CVD mortality. The impossibility of recognizing direct relationships between total fats, SFAs, MUFAs and PUFAs intakes and CHD and CVD mortalities in different regions based on data from Table 4 is evident. Thus, in spite of the highest consumption of total fat in Greece, only a relatively low value of CHD and CVD mortality was documented. Similarly, the highest PUFAs intake in Austria did not correlate with the high number of CHD and CVD mortality.

**Figure 3:**
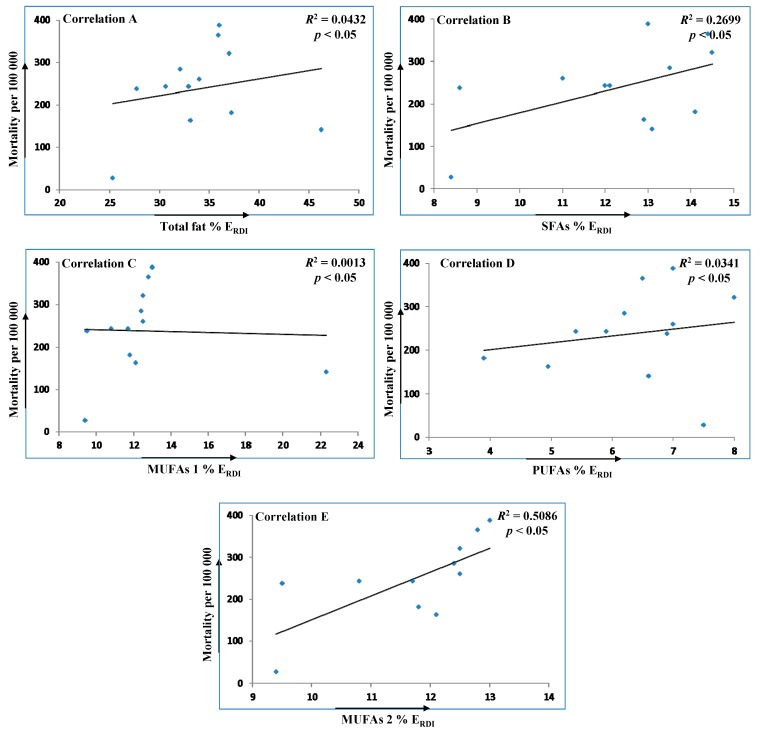
Spearman’s correlations illustrating relationships between CHD and CVD mortality per 100,000 people and intakes (% E_RDI_) of total fat (**A**); SFAs (**B**); MUFAs 1 (**C**); PUFAs (**D**) and MUFAs 2 (**E**) in various countries. MUFAs 1 is correlation including all countries, whereas MUFAs 2 is created without data from Greece.

In Figure 3, dependence of CHD and CVD mortality on total fats, SFAs, MUFAs and PUFAs intakes is demonstrated. In the case of MUFAs, two correlations were performed; in MUFAs 1 (correlation C) all monitored countries were included, while for MUFAs 2 (correlation E) it was created without data regarding Greece with a specific high consumption of olive oil, an abundant source of MUFAs. Statistical analyses using Spearman’s correlation have not confirmed the significant relationship between CHD and CVD mortalities and intakes (% E_RDI_) of total fats, SFAs, MUFAs 1 and PUFAs in various countries. However, providing data from Greece were excluded, correlation E between MUFAs 2 intake and mortality showed even higher significance than correlation between SFAs and mortality. It is evident that direct dependence between factors mentioned above does not exist. The impact of specific fatty acids on disease incidence is very difficult to explain because of numerous factors including lifestyle as well as various functions and relationships between individual fatty acids in human biochemical pathways.

Results of prospective epidemiologic studies have proved the correlation between specific types of fats and risk of CHD, but no relationships with total fats [20,62]. High level of CHD and CVD mortality in Western countries has provoked general nutritional recommendations to reduce dietary intake of SFAs as cardinal prevention of these diseases. However, conclusions from other studies have indicated that dietary SFAs reduction itself cannot suppress the risk of CHD and CVD incidence and mortality and do not clearly support cardiovascular guidelines promoting high consumption of n-6 PUFAs and suggest reduced consumption of SFAs [63,64]. A complex solution is needed. Mostly, reduction of SFAs and TFAs with their simultaneous replacement by PUFAs could lead to reduction of the risk of CHD [61]. However, some argue that impact of excessive consumption of n-6 PUFAs on risks of CVD leads to fixing an upper limit of ideally about 10% E [65]. In another systematic analysis focused on global, regional and national consumption levels of dietary fats and oils in 1990 and 2010, great differences in fatty acids dietary intakes were also stated [66].

Moreover, reduction of one macronutrient in the diet has to be accompanied by an increase of another macronutrient to maintain the energy balance. Mostly, replenishment of energy after a decrease of fat intakes is accompanied with an increase of carbohydrate intakes. However, higher sugar levels in food do not prevent the increasing trend towards obesity and the second type of diabetes. Short-chain SFAs, e.g., from dairy fat and coconut oil, can influence gene expression via interactions with various G-protein-coupled receptors responsible for several hormonal responses, including insulin and leptin regulating overall energy metabolism in the human body [41]. Low-fat, high-carbohydrate diets are known to reduce HDL cholesterol and to raise triglycerides associated with increased CHD risk [40,55]. Further, cohort studies have showed an influence of different dietary composition on CHD risk. Replacing SFAs with PUFAs was inversely associated with the risk of CHD, whereas replacing SFAs with carbohydrates was not associated with CHD risk [67].

What is more, the study of almost 80 thousand women performed age-analyses and body mass index-analyses and proved the inverse correlation between polyunsaturated fat intake and CHD risk, while the *trans*-fat intake was associated with the raised risk of CHD. The strongest association was among women younger than 65 and also in women with the body mass index higher than 25 kg/m^2^ [62]. Obesity and insulin resistance are both powerful predictors of CHD risk. Possibly, PUFAs can suppress the risk of CHD by improving both blood lipid levels and insulin resistance in overweight women, whilst TFAs may influence risk of CHD by its adverse effects on blood lipid levels [62,68].

Findings from many cohort studies are apparently inconsistent. Importantly, studies focused on the relationship between macronutrient intake and CHD have to be multifactorial as many environmental factors and personal conditions influence individual metabolic pathways. That is why the simple relationship between, *i.e.*, total fats, SFAs and PUFAs intakes and the risk of serious diseases could not be determined.

## 3. Experimental Section

### 3.1. Samples

The study was conducted on fourteen vegetable oils purchased in a specialized local store (Table 5).

**Table 5:** Description of analyzed samples of vegetable oils.

Oils	Samples	Plant Sources	Technological Process
Grape	GRP	*Vitis vinifera*	-
Peanut	PNT	*Arachis hypogaea*	-
Rapeseed	RPS	*Brassica napus*	Cold drawn
Sunflower	SFL	*Helianthus annuus*	Cold drawn
Safflower	SAF	*Carthamus tinctorius*	Cold drawn
Almond	ALM	*Amygdalus communis*	Cold drawn
Wheat germ	WHG	*Triticum aestivum*	Cold drawn
Sesame	SES	*Sesamum indicum*	Cold drawn
Pumpkin seed	PMS	*Cucurbita pepo*	Cold drawn
Rice brain	RB	*Oryza sativa*	-
*Silybum marianum*	SIL	*Silybum marianum*	Cold drawn
Coconut	COC	*Cocos nucifera*	-
Hemp	HMP	*Cannabis sativa*	Cold drawn
Olive	OL	*Olea europaea*	Cold drawn

### 3.2. Standards and Reagents

The standard mixture of 37 FAMEs (FAME Mix, SUPELCO, Bellefonte, PA, USA), and methyl-undecanoate (which was used as the internal standard) were purchased from Sigma Aldrich Chemical Co. (St. Louis, MO, USA). All other used chemicals were purchased from Merck (Darmstadt, Germany) and were of analytical purity.

### 3.3. Preparation of FAMEs

Firstly, fatty acids of oils have to be converted in their methyl esters (FAMEs) before gas chromatography (GC) analyses with a modification according to valid standard method in the Czech Republic [69] are performed. This method with usage of acid catalyst boron trifluoride is supported by the fact that basic catalysis does not convert free fatty acids especially in oils as can be concluded from the paper [70]. Briefly, 4 mL of 0.5 M sodium hydroxide in methanol were added to 1 mL oil sample, the equipment was closed and heated for 20 min under nitrogen. Further, 5 mL of 15% boron trifluoride, freshly prepared in methanol, was added to methylate the samples. After 2 min, 5 mL of heptane and 2 mL of saturated solution of sodium chloride were added and the sample was removed from the heating block (LTHS 250, Brnenska Druteva, Czech Republic). Then, 15 mL of heptane and 40 mL of saturated solution of sodium chloride were added to the extract of FAMEs, the mixture was shaken and phases were separated and washed subsequently with 40 mL of saturated solution of sodium chloride. Heptane phase was separated and anhydrous sodium sulfate was added. Prepared FAMEs were transferred into 50 mL volumetric flask together with 1 mL of methyl-undecanoate as the internal standard and replenished by hexan.

### 3.4. GC Analysis of FAMEs

Quantitative determinations of FAMEs were conducted according to [71] using a Shimadzu GC-2010 gas chromatograph (Shimadzu Corporation, Tokyo, Japan) with a flame ionization detector (FID) and capillary column HP-88 Agilent Technologies (100 m × 0.25 mm) with a stationary phase (88% cyanopropyl, aryl-polysiloxan) with the thickness of 0.2 μm. The injection volume was 1.0 μL, the temperature of injection port was 250 °C with the split ratio of 1:100 and nitrogen was used as a carrier gas, temperature program was 80 °C/5 min, 200 °C/30 min, and 250 °C/15 min. Identification of FAMEs was performed by comparing their retention times with those of reference standards (mixture FAME Mix, SUPELCO, which included 37 FAMEs). For quantification of FAMEs, methyl-undecanoate (Sigma Aldrich Chemical Co., St. Louis, MO, USA) was used as the internal standard. The results of FAs were expressed as percentages of total FAMEs.

### 3.5. Determination of Energy Contribution of Fatty Acid Groups of Vegetable Oils

Amounts of fatty acid contents (mg/g) were converted to energy values using the conversion factor of 37.7 kJ/g for fat and fatty acids, because of the evaluation of energy E (kJ/g oils) derived from SFAs, MUFAs, PUFAs, n-3 and n-6 PUFAs of analyzed vegetable oils [61].

Further, energy contribution E (% E_RDI_) of SFAs, PUFAs, n-3 PUFAs and n-6 PUFAs of analyzed oils to recommended dietary intakes for total fat (E_RDI_—37.7 kJ/g) was calculated and it was expressed as an amount (g) of analyzed oils needed to cover reported recommended daily intakes for individual fatty acid groups. Values of energy contribution of fatty acid groups of analyzed oils were calculated from the maximal values of recommended daily intakes (E_RDI_) according to [15] for fatty acids (E_RDI–SFAs_—10% E; E_RDI–PUFAs_—11% E; E_RDI–n-3_—2% E and E_RDI–n-6_—9% E).

### 3.6. Statistical Evaluation of Impact of Fat Dietary Intake on the Number of CHD and CVD Mortality in Various Countries

High number of CHD and CVD mortality in many Western countries is a serious problem caused by a complex of factors and the composition of dietary fat belongs to them. So, the evaluation of influence of dietary fats intakes on CHD and CVD mortality was also provided from the reported data of total fats, SFAs, MUFAs and PUFAs intakes among adults in different countries. Spearman’s correlations between SFAs, MUFAs and PUFAs intakes and CHD and CVD mortality were computed. The significance level was set to 0.05 (5%). Statistical analyses were conducted using QCExpert 3.3, TriloByte statistical software, Ltd. (Pardubice, Czech Republic).

## 4. Conclusions

A direct relationship between diet and human health was postulated by Hippocrates in the 4th BC. Energy balance is an important factor to maintain healthy body weight. Oversized intake of energy without adequate physical exertion leads to obesity and relating ailments that can result in serious diseases including CHD.

Vegetable oils are sources of important fatty acids, embracing all groups of SFAs, MUFAs, and PUFAs. However, their fatty acid composition varies according to the source plant or it depends on the technology process during their production. The study was focused on the oils obtained mostly by mechanical ways under the conditions that do not cause any changes in their chemical composition. From these results, it could be concluded that the majority of analyzed vegetable oils is characterized by higher amount of PUFAs, with especially higher amounts of n-6 PUFAs, so they could not be used as oils for daily consumption, but rather as oils to supplement specific PUFAs. The majority of them exceeded recommended intake of 11% E_RDI_. The highest contributions of PUFAs derived from 1 g oils were 786.8% E_RDI_ and 626.3% E_RDI_ in sunflower oil (SFL) and safflower oil (SAF), respectively. Thus, to cover recommended daily energy intake of PUFAs only 0.1 g of sunflower oil and 0.2 g of safflower oil, grape oil, *silybum marianum* oil or hemp oil is needed. These oil amounts were calculated in relation to the maximal values of recommended daily energy intakes for PUFAs.

Statistical analyses using Spearman’s correlation has not confirmed any direct significant relationship between CHD and CVD mortalities and intakes of total fats, SFAs, MUFAs and PUFAs in various countries. Studies focused on the relationship between total fats, SFAs and PUFAs intakes and CHD have to be multifactorial because the presence of many environmental factors and personal conditions that have simultaneous impacts on the human metabolic pathways.
